# Christopher Columbus

**Published:** 1892-10

**Authors:** 


### CHRISTOPHER COLUMBUS.

The discovery by the Superintendent of the Military Archives at Madrid of documents, probably setting at rest the doubts that formerly existed as to the birthplace of Columbus, must have awakened new interest in the history of the most renowned discoverer of the past. It is to be noted, however, that the documents only affirm tradition, for Genoa has always been the Admiral's accredited birthplace. But if the discovery should lead to nothing but a more careful investigation of the records of his later history, it will have been of use.

The character of Columbus has been greatly misunderstood, and his 600 biographers have in turn invested him with the glory of the religious hero and the contumely of the ill-tempered and crack-brained adventurer. An impartial critic must admit, indeed, that he was something of both, though more of the hero than the adventurer, and that his biographers have erred considerably in what Mr. R. L. Stevenson would call their "point of view."

Educated as it is supposed, in the local schools of Genoa, and for a short period at the University of Pavia, the youthful Columbus must have come in close contact with the scholars of the day. Naturally of a religious temperament, the piety of the learned would early impress him, and to this may possibly be attributed the feeling that he had been divinely selected, which remained with him until his death.

There is little doubt that he began his career as a sailor at the age of 14, with the sole object of plunder. The Indies were the constant attraction for the natives of Venice and Genoa; the Mediterranean and the Adriatic were filled with treasure ships. In these circumstances it is not to be wondered that the sea possessed a wonderful fascination for the youth of those towns. This opulence was the constant envy of Spain and Portugal, and Columbus was soon attracted to the latter country by the desire of Prince Henry to discover a southern route to the Indies. It was while in Portugal that he began to believe that his mission on earth was to be the discoverer of a new route to the land of gold—"the white man's god." For ten years he resided in Lisbon, from time to time making short voyages, but for the most part engaged drawing maps to procure himself a living. Here he married, here his son Diego was born, and here his wife, who died at an early age, was buried.

Toscanello at this time advanced the theory that the earth was round, and Columbus at once entered into correspondence with him on the subject, and was greatly impressed with the views of the Florentine scientist, both as to the sphericity of the world and the wonders of the Asiatic region. Heresy hunting was then a favorite pastime, and Columbus in accepting these theories ran no small risk of losing his life. Portugal and France in turn rejected his offers to add to their dependencies by his discoveries ; and, though his brother found many in England willing to give him the necessary ships to start on his adventures, Spain, after much importuning on the part of the explorer, forestalled that country.

Then followed his four eventful voyages with all their varying fortunes, and his death, when over 70 years of age, in a wretched condition of poverty. The ready consideration of theories, not only dangerous, but so astounding in their character as to throw discredit on those who advanced them, shows him to have been a man of intellectual courage. Humility was another trait in his character, and in all his life it cannot be said that he acted in any but an honest and straightforward manner toward his fellow men.

It is true, no doubt, that his recognition of slavery somewhat dims his reputation. He sold many Indians as slaves, but it should be remembered that slavery prevailed at the time, and it was only on his second voyage, when hard pressed for means to reimburse the Spanish treasury for the immense expense of the expedition, that he resorted to the barter in human flesh. Indeed, his friendly relations with the natives show that as a rule he must have treated them in the kindly manner that characterized all his actions.

Throughout the reverses of his long career, whether received with sneers, lauded as a benefactor of his country, put in chains by crafty fellow-subjects, or defrauded by an unscrupulous prince of the profit of his discoveries, he continued a man of an eminently lovable character, kind to his family, his servants, and even his enemies. Americans are to do honor at the Columbian Exhibition to the name of him who, though not the first white man to land on the shores of the new world, was the first to colonize its fertile islands. Not only America, but the whole world, may emulate his virtues with advantage ; for, even now, justice and mercy, courage and meekness, do not always abide together.

---

**Avoid** going into the presence of any contagious disease when perspiring or when the system is not properly fortified by food. An empty stomach and open pores increase the susceptibility to take the disease.

